# A Case Report of a Child With Attention Deficit and Hyperactivity Disorder With Persistent Acral Swelling and Desquamation Following Methylphenidate

**DOI:** 10.1192/bjo.2026.11901

**Published:** 2026-06-30

**Authors:** Sena Uzunay Ozsoy, Tuba Mutluer, Secil Vural

**Affiliations:** 1 Surrey and Borders Partnership Trust, Epsom, United Kingdom; 2 Koc University Hospital, Istanbul, Turkey; 3 Akdeniz University Hospital, Antalya, Turkey; 4 Halic University Hospital, Istanbul, Turkey

## Abstract

**Aims:**

Methylphenidate is a relatively safe medication used frequently in ADHD with only a few dermatological side effects reported up-to-date. While direct relation between the medication and the dermatologic side effects on children are discussed and supported by the disappearance of reactions after medication withdrawal, other confounding factors such as parental stress and coping mechanisms, were indeed observed to have a direct impact on children’s wellbeing. The higher the maternal anxiety was related to the worse outcome in children with stress. This fact could explain recently emerged or prolonged skin reactions especially during the pandemic, as it provokes health-related anxiety, especially in vulnerable people.

**Methods:**

Our case is a 9-year-old boy with ADHD who experienced digital desquamation after using methylphenidate-containing prescriptions. To the best of our knowledge, he was the first child having persistent non-pruritic, non-painful acral swelling and desquamation without any benefit from discontinuation of medications containing methylphenidate, and without any rheumatological condition.

**Results:**

There have been a few unusual adverse skin reactions to Methylphenidate reported, differing widely from general to local, pruritic to non-pruritic, and exfoliative to non-exfoliative. Our case had local non-pruritic exfoliation started 2 months after initiation of Methylphenidate -IR, however, the symptoms did not disappear after the withdrawal of prescriptions.

Persistent peeling after discontinuation of any Methylphenidate prescription was a unique situation. Thus, we investigated whether Methylphenidate has triggered and/or masked any rheumatologic condition especially juvenile rheumatoid arthritis and juvenile scleroderma. He was examined by a paediatrician with no anomaly found. Another reason for such prolonged skin reaction could be personal and parental stress. This could also explain why he has abdominal pain triggered only by going to school, as it may be related to parental expectations in addition to his ADHD and dyslexia comorbidity. The literature findings support the idea that high maternal anxiety elicits worse health outcomes in children with a neurodevelopmental disorder. Unfortunately, we could not have a chance to assess the anxiety level and coping mechanisms of the mother of our case, other than observing her stress-related behaviours as frequent doctor visits, to have a more concrete debate.

**Conclusion:**

Any skin finding after methylphenidate prescription must be monitored closely to have early preventive measures, better adherence to treatment, and quality of life for both patients and their parents. Also, both children and their parents could be controlled for stress and coping mechanisms to diminish their negative effects on dermatological or any other health-related outcome.

